# 

**Published:** 1984-01

**Authors:** 


JANUARY 1984
VOLUME 99 (i)
NUMBER 369
Published Quarterly
Annual Subscription £8 Post Free

### BRISTOL MEDICO-CHIRURGICAL JOURNAL

Established 1883

### BRISTOL MEDICO-CHIRURGICAL JOURNAL

Incorporating the Medical Journal of the South West

34. Correspondence

35. Miscellanea

36. Examination Results

Printed in Great Britain by John Wright & Sons (Printing) Ltd, at The Stonebridge Press, Bristol

### Editorial Committee

**EDITOR**
*Mr. M. G. Wilson*
*Litfield House, Clifton, Bristol BS8 3LS*

ASSISTANT EDITOR
*Dr. C. R. Tribe*
*Southmead Hospital, Bristol BS10 5NB*

MEMBERS
*Dr. M. B. Lennard*
*Dr. D. W. Wright*
*The Hon. Treasurer of the Society*
*The Hon. Secretary of the Society*

SECRETARY
*Mr. B. P. Jones, The Library, Medical School, University Walk, Bristol BS8 1TD*

### Correspondents

*Dr. J. Jancar*
*Dr. H. G. Mather*
*Professor G. M. Stirrat*
*Dr. J. L. G. Thomson*
*Dr. C. R. Tribe*
*Dr. M. J. Whitfield*
*Professor R. C. N. Williamson*
*Dr. D. W. Wright*

### Bristol Medico-Chirurgical Society

PRESIDENT 1983/84
*Dr. N. C. Tricks*

PRESIDENT ELECT

*Dr I. S. Bailey*

HONORARY SECRETARY
*Dr. H. M. Norman*
*Hillside, Vicarage Lane, Barrow Gurney*

HONORARY TREASURER
*Dr. J. B. Penry.*
*Southmead Hospital, Bristol BS10 5NB*

The Society was founded in 1874. In 1883 publication of the Journal began and has continued quarterly ever since then. During the years 1949 to 1962 it appeared under the title of 'The Medical Journal of the South West'.

### Notice to Contributors

*The Journal is especially concerned to provide a place for the recording of medical thought, interests and practice in and around Bristol. It therefore welcomes articles that, as well as being of immediate interest to members, will document the local and contemporary medical scene for our successors.*

*Original articles are preferred in the form proposed by the International Committee of Medical Editors (The Vancouver System) and published as an article in the British Medical Journal in 1979 (Br. Med. J.* **1***, 532–535). This has been reprinted as a pamphlet and may be obtained from the Editor of the B.M.J., B.M.A. House, Tavistock Square, London WC1H 9JR (enclose 50p).*

*Also welcome are letters to the Editor, Book Reviews, news of important appointments and events, etc.*

### University of Bristol Faculty of Medicine

**October 1983**

### THE DEGREE OF DOCTOR OF MEDICINE

David Nigel Fraser HARRIS
Christopher Julian PRITCHETT

### THE DEGREE OF DOCTOR OF PHILOSOPHY

John Stanley Hill GASTON
Nicholas Peter LUCKMAN

### THE DEGREE OF MASTER OF SCIENCE IN MEDICAL SCIENCES

Nithal Raouf MAHDI

